# Hypodermic Cathartics

**Published:** 1899-02

**Authors:** E. Stanton Muir

**Affiliations:** Instructor in Materia Medica and Pharmacy, Veterinary Department, University of Pennsylvania


### HYPODERMIC CATHARTICS.

By E. Stanton Muir, Ph.G., V.M.D.,
INSTRUCTOR IN MATERIA MEDICA AND PHARMACY, VETERINARY DEPARTMENT, UNIVERSITY OF PENNSYLVANIA.

(Concluded from page 35.)

### Barium Chloride.

Barium chloride is the third and last hypodermic cathartic that was used in this series of experiments. We administered the drug to five different animals, some in poor condition and others in good condition.

*Experiment No.* 24.—The first animal that received a dose of Barium chloride was a bay gelding, ten years old, 15¾ hands high. His general condition was poor; temperature 37.7° C. (100° F.). Pulse full and 60; respiration, 9; heart regular and strong. The solution used in these experiments was made as follows: Barium chloride (P. & W.), recrystallized, 20; distilled water, q. s. ft. 100, or a 20 per cent. solution. At 12.09, 1.0 of barium chloride in solution was thrown into the jugular; in eight minutes he defecated, the feces being normal. At this time we noted a very slight ptyalism, which would not have been noticed in any other animal. The second passage was made at 12.33, which was also normal. A third defecation at 12.47 was somewhat softer and of a larger quantity. At 2 P.M. the seventh and last feces was passed, there being no other defecation up to the end of the experiment, at 5.20 P.M. At no time was there the slightest evidence of pain. Amount of feces passed was a little over twenty pounds. There were no prominent symptoms except those of defecation.

*Experiment No.* 25.—Animal was a bay gelding, nine years old, 16¼ hands high. Temperature normal; pulse 48 and full; heart regular and strong. The general condition of this animal was poor. At 12.11, 2.0 of Barium chloride in solution were administered intravenously. In one minute there was a normal defecation, followed in two minutes by a second copious passage, which was softer. At 12.21 the respirations were 14; heart 42 and irregular, and liquid feces were voided. The twentieth passage was noted at 1.22, one hour and eleven minutes after administering the drug. All the passages up to this time were copious and gradually becoming more liquid. At this time the pulse was 48 and full, respirations 12, and the heart irregular. At 3.02 the twenty-fourth passage was made. During the experiment there was an almost constant passage of flatus and more or less mucus.

No more defecations were noted up to 5.20 P.M., at which time all abnormal symptoms were passed. Very slight if any pain was noted at any time during the experiment. Total weight of feces thirty-seven pounds and twelve ounces, and probably three-fourths of this amount was passed in the first one and one-quarter hours, during which time there were twenty defecations.

*Prominent symptoms* were those accompanying extreme purgation, and only slight pain.

*Experiment No.* 26.—Bay gelding, nine years old, 15¾ hands high; pulse 72 and full; heart rapid and irregular. General condition was good; temperature 38° C. (100.6° F.). This animal was used two months previous for experiments with arecoline hydrobromate.

At 12.16 the first intravenous injection of a solution containing 1.0 barium chloride was administered. At 12.19, or in three minutes, the horse passed a large quantity of feces of a normal consistence. At 12.25 he showed some slight signs of abdominal pain. At 12.28 second defecation, which was similar to the first. A slight ptyalism was noted at 12.30, but, like that in the animal used in Experiment 24, it passed off quickly. The temperature at 12.50 dropped to 37.6° C. (99.7° F.), and there was some sweating, more or less general. The third defecation took place at 1.01, of large quantity and softer. At 2.14 the fifth and last passage was noted, and, like the rest, was of good quantity and soft. At 2.25 all symptoms had passed off, except he was champing his jaws. Symptoms of distress were not extreme, but were more than in any of the other experiments with this drug, which we considered was due to faulty action of the heart, noted before the injection was made. There were no more passages up to 5.20 P.M. The feces voided weighed fourteen and one-half pounds, and I might here state that the collection and weighing were done with as much accuracy as possible.

*Prominent symptoms* were purgation, feces becoming quite soft, slight distress and colicky pains, some sweating, and slight ptyalism.

*Experiment No.* 27.—This I look on as the most interesting experiment during the course, on account of the peculiar action of the drug on the kidneys as well as that on the alimentary tract. The animal used was a roan gelding, aged, 15¾ hands high. Pulse 48 and strong; heart regular and impact good. The general condition of the animal was fair. At 2.13 a solution containing 2.0 of the salt was given intravenously. In one minute he defecated, the feces being normal in every way.

At 2.22 he had had six passages, the feces being accompanied with mucus. The heart became somewhat irregular, and he showed some signs of pain. At 2.26, thirteen minutes after giving the drug, he had his ninth passage, and from that time until 3.08 there were no more passages, nor was there any mucus running from the anus; but during that time, from 2.35 until 3.07, he passed his urine seven times, a large quantity on each occasion. The urine was very frothy. At 3.08 he passed one ball, and the same at 3.09, and up to 4.40 he had, all told, forty-two defecations, none of them large in quantity, but all soft, toward the last becoming quite liquid.

During the course of the experiment this animal micturated fourteen times, starting from the first at 2.35 and ending at 4.53. From this hour no urine nor feces was voided up to 5.20. He showed some signs of colicky pains throughout this experiment, but never severe enough to give us any concern. The total amount of feces passed weighed twenty-six pounds. The amount of urine passed was not known, as we did not expect such an action from the drug, and were not supplied with vessels to collect it, but I think it would have taken almost a half-barrel to hold it all. We noted the fact that as the quantity of voided urine increased the amount of feces became less.

*Prominent symptoms* were purgation, slight pain, micturition.

*Experiment No.* 31.—A bay gelding in fair condition was used in this experiment. He was twelve years old, 15.2 hands high, weighed 900 pounds. Temperature 37.2° C. (99° F.). Pulse 50, full and strong; heart irregular, but strong; respirations 12. We injected a solution containing 2.0 of the salt into the jugular at 11, and at 11.31 he had had eleven passages, some small. At the latter hour the pulse was full, strong, and 48, and the heart strong. At 12.07 he had defecated eighteen times, and watery mucus was constantly running from the anus. Being desirous of knowing the toxic amount of barium chloride, we gave at 1.43 2.0 more of the salt, dissolved in water, in the vein. At 1.48 he had had two more passages, making twenty since giving the first injection. At 1.49 there were spasmodic muscular movements, and he fell down, breathing became stertorous and gasping, and at 1.52 his heart ceased to beat, and he was over his troubles. The autopsy, which we held at once, revealed the following: Blood cyanosed, right heart empty, left side full, lungs normal, cæcum contents fluid, intestines full of gas, but little feces; folded colon much congested; lower bowels full of feces; small intestines and mucous membrane normal; cause of death, heart failure.

*Prominent symptoms* were extreme purgation, voiding of watery mucus, only slight pain until second dose of 2.0 was administered, after which all the symptoms of asphyxia prevailed, ending in the death of the animal in nine minutes after the second dose of 2.0 was given.

| Exp. No. | Animal. | Drug used. | Dose. | Mode of administration. | Repeated. | Time to first symptoms. |
|---|---|---|---|---|---|---|
| 1 | G.G. | Pysostig. sulph. | 0.1 (1½ gr.) | Subcutaneous. | No. | 10 minutes. |
| 2 | G.G. | " " | 0.1 (1½ " ) | Intravenous. | " | 7 " |
| 3 | G.G. | " " | 0.2 (3 " ) | Subcutaneous. | Once. | 32 " |
| 4 | G.G. | " " | 0.2 (3 " ) | Intravenous. | No. | 7 " |
| 20 | G.G. | " " | 0.1 (1½ " ) | Subcutaneous. | " | 11 " |
| 9 | G.G. | Arecöl. hydrobr. | 0.05 ( ¾ " ) | Intravenous. | Once. | 2 " |
| 10 | B. G. | " " | {3 doses 0.2 (0.3 " )} | Subcutaneous. | Twice. | 6 " |
| 28 | B. G. | " " | 0.01 (0.15 " ) | Intravenous. | No. | 1 " |
| 29 | B. G. | " " | {0.2 (0.3 " ) 0.01 (0.15 " )} | " | Yes. | 2 " |
| 30 | B. G. | " " | 0.02 (0.3 " ) | " | No. | 7 " |
| 24 | B. G. | BaCl | 1.0 (15.4 " ) | " | " | 8 " |
| 25 | B. G. | " | 2.0 (30.8 " ) | " | " | 1 " |
| 26 | B. G. | " | 1.0 (15.4 " ) | " | " | 3 " |
| 27 | B. G. | " | 2.0 (30.8 " ) | " | " | 1 " |
| 31 | B. G. | " | 2.0 (30.8 " ) | " | Once. | 9 " |

We can, I think, arrive at the following conclusions:

1. Barium chloride in doses of 2.0 may be safely administered intravenously.
2. It is a prompt and certain cathartic in doses of from 1.0 to 2.0 gms.
3. Its administration is not followed by any severe pain or annoyance to the animal.
4. It acts quickly if there is no mechanical obstruction of the bowels, such as a calculus or volvulus, even in the latter case it may cause it to untwist.
5. It has the advantage of being cheap.
6. In doses of 1.0 it causes no pain, while if 2.0 be administered intravenously there are some slight signs of pain and distress.
7. It causes little if any sweating, and no reduction in temperature.
8. Although the number of defecations may be small, the average amount of feces passed is large.

SUMMARY.—We find, on consulting the table, that Barium chloride acts quickly, in from one to nine minutes, causing large quantities of feces to be voided, but does not cause pain, and the action ceases in from two to three hours; that Physostigmine sulphate causes abdominal pain, is longer producing evacuation of the bowels, and its action lasts from two to four hours, causing the expulsion of large quantities of feces, mucus, and flatus; that

| First symptom. | First defecation. | No. defecations. | Weight of feces. | Time elapsed. | Most dangerous symptoms. |
|---|---|---|---|---|---|
| Abdominal pain. | 38 min. | 11 | 10 lbs. | 2.16 | Colic. |
| Muscular twitching. | 11 " | 16 | 20 " | 1.54 | None. |
| Defecation. | 32 " | 25 | 17 " | 3.05 | Muscular weakness. |
| Abdominal pain. | 12 " | 38 | 28 " | 2.46 | " " |
| Pain; defecation. | 11 " | 12 | | 1.05 | Colic. |
| Ptyalism; muscular weakness. | 4 " | 15 | | 3.00 | Loss of coördination. |
| Ptyalism. | 64 " | 7 | | 10.45 | " " |
| Ptyalism; muscular weakness. | 6 " | 5 | 8¼ " | 5.00 | " " |
| " " " | 3 " | 6 | 12¼ " | 5.00 | " " |
| Slight twitching. | 8 " | 12 | 8 " | 0.50 | " " |
| Defecation. | 8 " | 7 | 20 " | 1.51 | None. |
| " | 1 " | 24 | 37¾ " | 2.51 | " |
| " | 3 " | 5 | 14½ " | 2.00 | " |
| " | 1 " | 42[1] | 26 " | 2.40 | " |
| " | 9 " | 20[2] | | 1.48 | Spasm of heart. |

Arecoline acts quickly, but causes more pain than either of the other salts used—its action lasts longer, not passing off in less than about five hours, and the amount of feces voided is small and never very soft, but it produces a profuse flow of saliva, and there is always an involuntary dripping of urine.

